# Rodent Arena Tracker (RAT): A Machine Vision Rodent Tracking Camera and Closed Loop Control System

**DOI:** 10.1523/ENEURO.0485-19.2020

**Published:** 2020-05-12

**Authors:** Jonathan Krynitsky, Alex A. Legaria, Julia J. Pai, Marcial Garmendia-Cedillos, Ghadi Salem, Tom Pohida, Alexxai V. Kravitz

**Affiliations:** 1 National Institute of Diabetes and Digestive and Kidney Diseases, National Institutes of Health, Bethesda, MD 20892; 2 Signal Processing and Instrumentation Section, Office of Intramural Research, Center for Information Technology (CIT), National Institutes of Health, Bethesda, MD 20814; 3 Departments of Psychiatry, Anesthesiology, and Neuroscience, Washington University in St. Louis, St. Louis, MO 63110; 4 Department of Neuroscience, Washington University in St. Louis, St. Louis MO 63110

**Keywords:** machine vision, mouse, rodent, tracking, video

## Abstract

Video tracking is an essential tool in rodent research. Here, we demonstrate a machine vision rodent tracking camera based on a low-cost, open-source, machine vision camera, the OpenMV Cam M7. We call our device the rodent arena tracker (RAT), and it is a pocket-sized machine vision-based position tracker. The RAT does not require a tethered computer to operate and costs about $120 per device to build. These features make the RAT scalable to large installations and accessible to research institutions and educational settings where budgets may be limited. The RAT processes incoming video in real-time at 15 Hz and saves *x* and *y* positional information to an onboard microSD card. The RAT also provides a programmable multi-function input/output pin that can be used for controlling other equipment, transmitting tracking information in real time, or receiving data from other devices. Finally, the RAT includes a real-time clock (RTC) for accurate time stamping of data files. Real-time image processing averts the need to save video, greatly reducing storage, data handling, and communication requirements. To demonstrate the capabilities of the RAT, we performed three validation studies: (1) a 4-d experiment measuring circadian activity patterns; (2) logging of mouse positional information alongside status information from a pellet dispensing device; and (3) control of an optogenetic stimulation system for a real-time place preference (RTPP) brain stimulation reinforcement study. Our design files, build instructions, and code for the RAT implementation are open source and freely available online to facilitate dissemination and further development of the RAT.

## Visual Abstract

**Figure:**
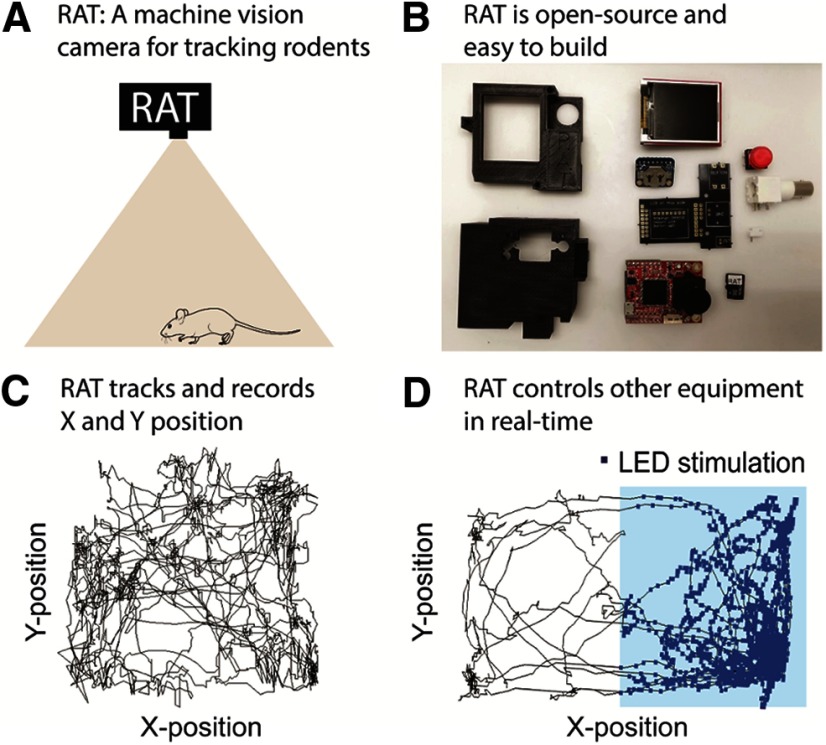

## Significance Statement

Video tracking is a critical tool in behavioral research. Here, we present an open source machine vision tracking device called the rodent arena tracker (RAT). The main advance of our device over what has been previously done with rodent video tracking is that our solution is small and battery powered, versus a tethered computer running a software package. This small form factor (about the size of a point-and-shoot camera) can enable new uses for video tracking, including in places where traditional video tracking solutions would be cumbersome or not possible.

## Introduction

Video analysis has greatly improved animal behavior monitoring methodologies since its first application in research. In early uses of this technology, human observers watched saved videos and manually quantified the frequency or patterns of various behavioral events. Advances in computer vision led to the development of algorithms that automatically segment video frames and track rodent position across time. Multiple open-source and commercial solutions followed this technological progress (Noldus et al., 2001; Tort et al., 2006; Aguiar et al., 2007; Patel et al., 2014; Lopes et al., 2015; Salem et al., 2015; Samson et al., 2015; Ben-Shaul, 2017; Poffé et al., 2018; Rodriguez et al., 2018; Pennington et al., 2019; Shenk, 2019). More recent advances in machine vision and imaging sensors have enabled automatic identification of behaviors and tracking of specific body parts such as limb or whisker movements (Hong et al., 2015; Huang et al., 2015; Wiltschko et al., 2015; Reeves et al., 2016; Nashaat et al., 2017; Mathis et al., 2018; Salem et al., 2019). Although many groups have developed methods to track rodents via video, with the exception of Nashaat et al. (2017), prior approaches all require a tethered computer for computation, and some require post-recording analysis due to high computational load of the processing applications. Such implementations can limit flexibility and scalability for high throughput experimental installations.

To circumvent these limitations, we developed the rodent arena tracker (RAT), which is capable of automatically tracking mice in high contrast arenas and using position information to control other devices in real time. Here, we present the design files, software, and validation and testing data to demonstrate the utility of the RAT. While rodent tracking has been accomplished by multiple other systems and corresponding software packages (as referenced above), the RAT device offers several novel and useful features, including: (1) onboard processing with no requirement of a connected computer, simplifying experimental pipelines; (2) battery powered option for wireless use; (3) reduced data storage needs afforded by real-time video processing; (4) low cost of ∼$120 per device; and (5) open-source implementation facilitating experiment reproducibility in other laboratories, as well as future method development.

As proof of concept, we implemented a dynamic thresholding algorithm that is effective at tracking rodents in high contrast arenas. The code is open-source, and the OpenMV camera provides additional libraries to enable more elaborate vision algorithms. Therefore, researchers can develop more elaborate processing methods with this same hardware to address their specific research problems. We also perform three practical use-case studies to demonstrate the utility and capabilities of the RAT in a research setting.

## Materials and Methods

### OpenMV camera tracking implementation

The RAT acquisition and real time processing software was programmed in the OpenMV integrated development environment (IDE). The image is processed using the following steps: (1) an image is acquired and saved to a frame buffer; (2) the image is segmented using a dynamic thresholding procedure; (3) contiguous “blobs” of pixels in the image are filtered based on a minimum and maximum size threshold and the centroid information for the largest valid blob is retained as the mouse centroid data; (4) mouse speed is computed using the inter-frame centroid difference; (5) the centroid of the mouse position and speed and positional data are overlaid on a feedback image on the LCD screen; (6) the RAT obtains the current date and time from its onboard real-time clock (RTC) module; and (7) data are locally stored in a text file including a per-frame timestamp, centroid values, and computed speed value. In addition to this processing scheme, the dynamic segmentation threshold is updated every 50 frames (∼4 s) to automatically adjust for potential changes in lighting. Added device functionalities for validation experiments included logging of Transistor-Transistor Logic pulses from an external device on the RAT input/output pin and triggering of an external device from the RAT input/output pin.

### Design

**Figure 1.**
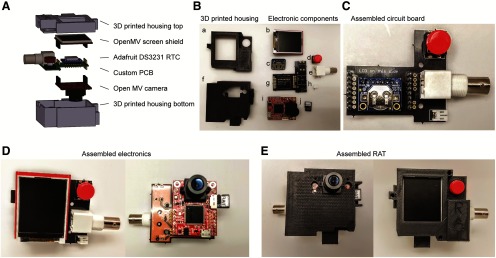
Assembly of the RAT. ***A***, Exploded schematic of the major parts for building RAT. ***B***, Photograph of the parts for building rat. ***a***, 3D-printed housing top. ***b***, OpenMV LCD shield. ***c***, Adafruit DS3231 RTC module. ***d***. Push-button. ***e***. BNC connector. ***f***, 3D-printed housing bottom. ***g***, Custom PCB. ***h***, JST 2 pin connector. ***i***, OpenMV M7 camera. ***j***, MicroSD card with RAT code. ***C***, Photograph of assembled RAT circuit board. ***D***, Photograph of the assembled RAT electronics. ***E***, Photograph of assembled RAT in 3D-printed housing.

The most important component of the RAT hardware is the OpenMV Cam M7 (available at https://openmv.io) which acquires and processes images to extract mouse location data (Fig. 1). The OpenMV Cam M7 also has built-in near-infrared (NIR) LEDs which are always on to enable illumination and tracking in dark environments. We designed a printed-circuit board (PCB) with a battery connection, BNC output, header for attachment of an Adafruit RTC module, and push-button for controlling the RAT, as well as a 3D-printed housing (Fig. 1). The RAT can be powered with an external battery, or via its micro USB port. All design files necessary to complete this build (including electronic layout/soldering instructions, Python code, and 3D printing design files) are located at https://hackaday.io/project/162481-rodent-arena-tracker-rat (also see Table 1).

### Build instructions

RAT device fabrication, assembly, and programming are outlined at https://hackaday.io/project/162481-rodent-arena-tracker-rat, including a step-by-step assembly video. We estimate that assembling the RAT takes ∼90 min. To assemble the hardware for the device, first populate the breakout PCB by soldering the tactile button, right-angle BNC connector, JST right-angle connector, and long male headers to the top of the board. Solder the included headers to the OpenMV Cam M7 with the female pins facing away from the side with the lens. The male pins of the headers should be trimmed using wire cutters so they do not exceed the height of the other components on the OpenMV Cam M7. Finally, solder the RTC module directly onto the PCB using including male headers, with the battery holder facing toward the LCD shield (it is positioned this way for easy removal of battery if necessary; Fig. 1*A–C*). Once the breakout PCB and OpenMV Cam M7 are assembled, mount the OpenMV Cam M7 in the bottom of the 3D-printed enclosure (Fig. 1*D*,*E*). The lens will fit through the square opening at the bottom of the enclosure, and the two mounting holes on either side of the OpenMV Cam M7 will align with their counterparts on the 3D-printed enclosure. Secure the OpenMV Cam M7 to the 3D-printed enclosure using a 4–40 screw in each of the two mounting holes. Connect the breakout PCB to the mounted OpenMV Cam M7 by aligning the mating faces of the connectors and pushing them together until they’re fully engaged. After the headers are connected, secure the breakout PCB to the enclosure using a 4–40 screw through each of the two mounting holes on the breakout PCB. Plug the LCD shield into the top of the breakout PCB by aligning the pins on the shield with the header rows on the breakout PCB. Next, align and mount the top cover of the 3D-printed enclosure with the base using 4–40 screws in each mounting hole. Finally, unscrew the supplied camera lens from the OpenMV camera, remove the small IR optical filter from the back of the lens with forceps, and replace the lens on the camera.

### Programming the RAT device

To program and configure the RAT, first download and install the OpenMV IDE (https://openmv.io/pages/download) and download the two files, RAT_v1.1_setTime.py and RAT_v1.1_auto_threshold_RTC.py from the project’s hackaday page (https://hackaday.io/project/162481-rodent-arena-tracker-rat). Format a microSD card as FAT32 and plug it into the RAT’s microSD card slot on the side of the enclosure. Open the OpenMV IDE on a PC, connect the RAT to the PC using the micro USB port on the back of the unit, and pair it with the IDE by clicking the connect button at the bottom of the IDE interface. Load “RAT_v1.1_setTime.py” in the OpenMV IDE and edit it to include the current date and time. Click the green arrow and it will program the RTC with the correct time. Once this is set it will not need to be reset for approximately five years, or until the coin cell in the RTC module dies. Next load “RAT_v1.1_auto_threshold_RTC.py” and navigate to Tools>Save open script to OpenMV Cam to upload the code. Unpair the RAT from the IDE using the disconnect button at the bottom left of the IDE and disconnect it from the PC. When using the device for the first time, focus the RAT’s lens using the live feed as a reference and lock it into place using the screw on the RAT’s lens holder. Take care not to overtighten this lens screw as it can easily break. The OpenMV Python files for controlling the RAT are also provided as Extended Data 1.

10.1523/ENEURO.0485-19.2020.ed1 Extended data 1 RAT_code.zip. Code to set the clock and run the tracking algorithm on the RAT. Download Extended Data 1, ZIP file.

### Operation instructions

Connect the RAT into a power source using either an external battery or micro USB cable. As soon as the device receives power it will create a new experiment data folder, begin tracking, and start recording data. The mouse centroid and speed will be overlaid on a feedback image on the LCD screen along with the current time and the experiment data file name. Press the button on the device to start a new data file. The new filename will appear on the screen and all rows in the data file will be time-stamped with the current date and time.

### Subjects for validation experiments

A total of ten adult male mice (nine C57Bl/6J black mice, one BALB/cJ white mouse) were housed in murine vivarium caging in a 12/12 h light/dark circadian cycle at room temperature. Four additional mice expressing D1-cre were obtained from the GENSAT project (EY242; Gong et al., 2007; Gerfen et al., 2013). Mice were given *ad libitum* access to rodent laboratory chow (5001 Rodents Diet; LabDiet) and water, and cages were changed every two weeks. Treatment and use of all animals conformed to the welfare protocols approved by the National Institute of Diabetes and Digestive and Kidney Diseases/National Institutes of Health Animal Care and Use Committee.

### Viral infusions and optic fiber implantation

Viral infections of DMS were conducted on four adult male mice (8–12 weeks old). Anesthesia delivered via a mouse mask mounted on a stereotaxic apparatus (Stoelting) was administered with isoflurane at 2–3% and maintained during the entire surgery at 0.5–1.5%. Ear bars secured the mouse head in place while the skin was shaved and disinfected with a povidone/iodine solution. The skull was exposed and a hole ∼0.5–1 mm in diameter was opened with a microdrill. A hydraulic injection system (NanoJect III) was loaded with AAV virus for expressing channelrhodopsin-2 in a cre-dependent manner (UNC viral core), and lowered into the brain at the following coordinates: AP +0.5 mm, ML ±1.5 mm, DV −2.8 mm (from bregma). A total volume of 500 nl of viral solution was delivered to each side of the brain, and the injector was left in place for 5 min after the infusion. In the same surgery, the mouse received two fiber optic cannulae (200 μm, 0.39 NA, 1.25 mm, ceramic ferrule) for optogenetic stimulation, secured to the skull with dental adhesive.

### Use case validation experiments

In experiment 1, the circadian study, a single C57NL/6J mouse was placed in a 9 × 12” Plexiglas box that was enclosed in a light-tight cabinet for 4 d, with *ad libitum* access to food and water. Lights were left off for the duration of the experiment. The RAT was positioned above the box for continuous tracking.

In experiment 2, four C57NL/6J mice were individually housed in 9 × 12” Plexiglas boxes with a FED feeding device (Nguyen et al., 2016) attached to the side and a RAT mounted above facing the arena floor. The output of the FED was connected to the input of the RAT, enabling the RAT to log the time and position of pellet retrieval events.

In experiment 3, four mice expressing channelrhodopsin-2 in direct pathway neurons and with unilateral optical fiber implants, were individually placed in a 9 × 12” Plexiglas box. The RAT device was centered over the Plexiglas box, and a 15-Hz triggering pulse was generated when the mice were detected in one side of the box. A wireless head-mounted LED stimulator (Plexon Helios) was placed on the head of each mouse, controlled by the pulses from RAT. The mice received unilateral stimulation when they entered one side of the box. After 15 min, the stimulation side was reversed.

### Software availability

All code and design files are freely available at https://hackaday.io/project/162481-rodent-arena-tracker-rat.

## Results

**Figure 2.**
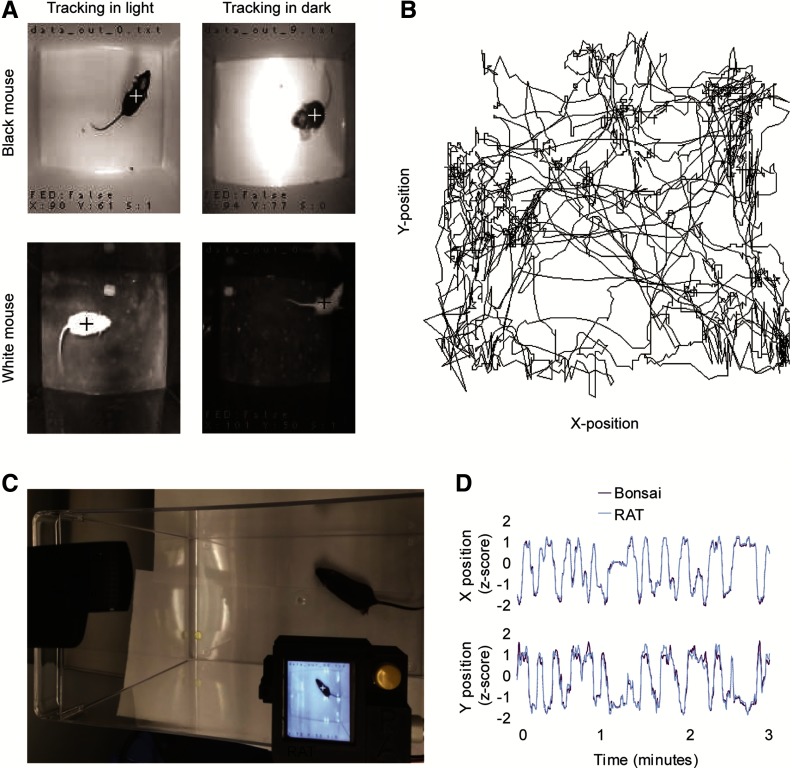
Validation of tracking performance. ***A***, Example images of black and white mice tracked by RAT in light or dark conditions. ***B***, Example *xy* scatter track plot of data exported from RAT. ***C***, Photograph of experimental validation setup recording the same mouse with RAT and a webcam connected to Bonsai. ***D***, *x* and *y* positions from both RAT and Bonsai, demonstrating strong correlation in mouse position data between the two systems.

We evaluated RAT performance under different lighting conditions using both black and white mice in a high contrast arena with the room lights on and off (Fig. 2*A*,*B*). The dynamic thresholding procedure was robust against changes in room lighting, automatically “re-thresholding” every ∼4 s to continue to track the mice. The RAT tracked black mice on a white background in both lighting conditions, although non-reflective flooring was necessary to limit the glare created from NIR LED reflections when tracking in the dark. We modified the segmentation code and threshold for tracking white mice on a black background and the device performance was comparable to the black mouse test (Fig. 2*A*,*B*). To validate the tracking performance, we compared the RAT data output head-to-head with video tracking in Bonsai, an open-source software language that is widely used for video tracking applications (Lopes et al., 2015). We positioned the RAT device and a USB camera connected to Bonsai above an arena containing a single black mouse (Fig. 2*C*). Both systems tracked mice successfully, with no instances of lost tracking. A quantitative analysis revealed 94.9% correlation between the *x* and *y* tracking positions of the RAT and Bonsai (Fig. 2*D*, *n* = 2 mice). We concluded that the RAT device was robust against changes in lighting and is useful for tracking mouse centroid position.

**Figure 3.**
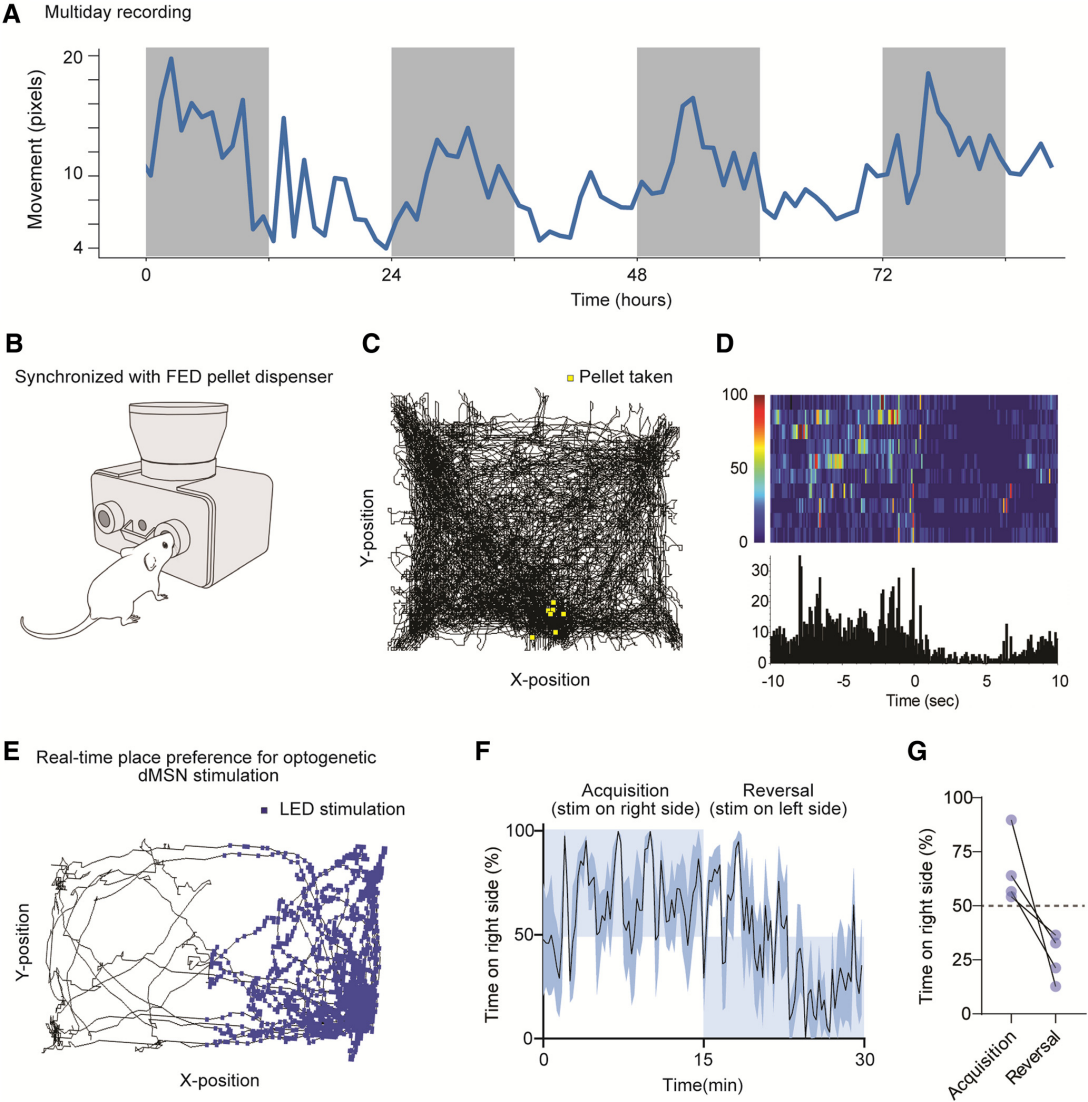
Experimental use cases for RAT. ***A***, Demonstration of RAT tracking, showing a circadian rhythm in movement levels over 90 h. Lights were off for duration of experiments, gray bars represent the normal night cycle. ***B***, Schematic of mouse nose-poking to obtain pellets from FED3 device. ***C***, Example track plot for 30 min, showing locations of a mouse when he retrieved pellets from FED3. ***D***, Perievent histogram and raster showing speed of mouse around pellet retrieval events. ***E***, Example track plot for optogenetic real-time self-stimulation experiment. Blue squares show location when blue LED turned on to stimulate direct pathway medium spiny neurons in the striatum. ***F***, ***G***, Quantification of average time on each side of the chamber (*n* = 4 mice).

In addition to validating RAT tracking with two mouse coat colors and two lighting conditions, we performed three experiments to demonstrate device utility and evaluate how the RAT performed in real-world “use-cases.” In experiment 1, we assessed how the RAT would perform in a multi-day circadian study. We positioned the RAT over a single mouse in a dark chamber for ∼3.5 d (90 h). As the RAT does not save video, this experiment generated a single text file that was ∼100 MB in size, which we estimated to be ∼20–100 times smaller than a video stream of the same length. The circadian rhythm of mouse activity was apparent in the RAT data, even in total darkness, demonstrating the utility of RAT for measuring endogenous circadian activity rhythms (Fig. 3*A*).

In experiment 2, we synchronized the RAT input/output connection with an open-source pellet dispensing device, the feeding experimentation device (FED; Nguyen et al., 2016). We programmed the FED device to send a TTL pulse to the RAT each time a pellet was taken (Fig. 3*B*). We individually tested four mice in this setup, enabling us to synchronize mouse activity with pellet retrieval. We recorded both the position of the mouse at the time of pellet retrieval and the speed of the mouse around these events (Fig. 3*C*,*D*).

Finally, in experiment 3, we re-programmed the input on RAT to operate as an output for a real-time place preference (RTPP) brain stimulation study. We expressed an excitatory opsin, channelrhodopsin-2 in direct pathway neurons in the striatum, a population of neurons that is reinforcing when stimulated (Kravitz et al., 2012). When the mouse crossed onto one half of the box, the RAT sent 15-Hz TTL pulses to a wireless transmitter that delivered 4-mW pulses of blue light to the mouse. This stimulation was highly reinforcing, resulting in rapid acquisition of preference behavior toward the LED-paired side of the cage within 5 min of the first session (*n* = 4 mice; Movie 1; Fig. 3*E*,*F*). After 15 min, we reversed which side was stimulated by rotating the RAT camera 180 degrees. This reversal caused the mice to rapidly switch their preference to the opposite side (Fig. 3*F*,*G*). As both the RAT and the optogenetic stimulation device were wireless, this experiment highlighted the simple and flexible nature of embedded electronics for research applications.

Movie 1. RTPP_example.mp4. Video demonstrating the real-time-place-preference assay. 10.1523/ENEURO.0485-19.2020.video.1

## Discussion

### Review of the device

The RAT is a low cost, wireless position tracker, optimized for tracking mice in high contrast arenas. The RAT is based on the OpenMV Cam M7 (openmv.io), an open source machine vision camera. We optimized control code for tracking mice and created a hardware board for conveniently connecting a battery, RTC, BNC input/output, and push button for starting the recording. We present validation data demonstrating the effectiveness of the device for tracking mice, as well as connecting the RAT to other devices for flexible experimental arrangements.

### Comparison with current technologies

Many commercial and open-source solutions exist for video tracking of rodents, and they all achieve high accuracy detection. Nearly all also have a richer feature set than the RAT and can accomplish more complex tracking and behavioral control tasks, including importing diverse data types and task control. As a pure tracking solution however, we see the value of RAT in its compact form factor, simplicity, and low cost.

### Device limitations

There are several limitations to the RAT. The first is it does not save video. Because of the size of the OpenMV Cam M7 frame buffer and the real-time video processing, it was not possible for the hardware to also save video. This limitation means experimental videos cannot be “re-scored” at a later date. The consequence is that more up-front testing is required to ensure the tracking algorithm is working before use in experiments. In our hands, the RAT works consistently and accurately on rodents in high contrast environments, and we noted no dropped data points in validation testing. We recommend that new users test in their own environments as changes in camera position or lighting could require modification to the tracking settings. A second limitation is the RAT does not have any automated calibration procedure for measuring the size of an arena. Currently, tracking data must be calibrated off-line to get real-world position and speed values (i.e., in centimeters and centimeters per second). While this process could be implemented onboard on the RAT, it would likely be cumbersome on the small device. Finally, data are saved to an on-board microSD card which must be removed to retrieve the data. In future versions of the RAT, we hope to include wireless communication technology that will stream tracking data in real time. Wireless data transfer will be especially important in large installations where removal of many SD cards would be cumbersome.

### Potential future improvements

We envision several future improvements that can be made to both the hardware and the software of the RAT. The OpenMV project is actively developing new hardware to increase processing power and memory of the camera, allowing for more advanced algorithms to run in real time. For example, while this paper was in review the OpenMV project released the OpenMV H7 model, which is faster and more powerful than the M7 model used here. Our code and hardware are forward-compatible with the H7 camera, which should be able to achieve higher frame rates for tracking. In addition, the OpenMV project is actively supporting new camera sensors, including an infrared heat sensor for tracking heat signatures, which may be useful for improving tracking and identification of specific behaviors. Additionally, the OpenMV camera uses the common M12 lens mount, enabling use of many commercially available lenses and optical components. Tracking algorithms may have to take the specific lens being used into account, particularly if it distorts the image geometry, as with a fish-eye or super-wide-angle lens. As the OpenMV hardware improves, the camera board in the RAT can be upgraded to enable new functionality.

We prioritized low rates of data storage by tracking in real-time and storing only tracking positions and speed. This low data rate should also be compatible with wireless data transfer. The OpenMV project already sells a WIFI-enabled “shield” for OpenMV cameras, and there is discussion online that a Bluetooth shield is being developed. Because of the low data rate, tracking data from multiple RAT devices could be sent automatically to an Internet server for remote monitoring of tracking data. Additionally, the existing data storage method could be changed to a more compressed format such as a binary data file to further reduce bandwidth and storage requirements.

Finally, the hardware presented here is limited to a single input/output pin, which is tied to the single analog output pin of the OpenMV camera. This allows for a user to export a derived parameter such as speed in real time. In future versions of the RAT, we hope to include more digital inputs and outputs to create richer interactions between the user, the RAT, and additional external devices. These examples for improvement are not exhaustive, and we imagine that individual users will have diverse and specific modifications. The open-source nature of the RAT allows researchers to modify functionality to fit their specific needs. We put all the code and design files we produced online, where we will include further modifications as they are developed.

## Conclusion

The RAT is a machine vision tracking device based on the OpenMV Cam M7. The RAT is wireless, inexpensive, and offers real-time processing and low storage requirements, all of which facilitate large-scale studies of animal behavior. Open-source implementations like this enable experimental reproducibility across research centers and can lead to innovative new rodent-based experiment methodologies.

**Table 1:** Bill of materials

Component	Number	Cost per unit	Total cost	Source of materials
OpenMV Cam M7	1	$65.00	$65.00	Openmv.io
LCD shield	1	$20.00	$20.00	Openmv.io
Adafruit DS3231 RTC breakout	1	$13.95	$13.95	Adafruit.com
3D-printed enclosure	1	∼$5	∼$5	Any printer will work
Breakout PCB	1	$2.00	$2.00	Seeed.io
JST right-angle connector	1	$0.95	$0.95	Karlsson Robotics P/N PRT-09749
Tactile button	1	$0.49	$0.49	Karlsson Robotics P/N COM-10302
Long break away male headers	2	$0.75	$0.75	Mouser P/N 474-PRT-12693
Right-angle BNC connector	1	$2.43	$2.43	Mouser P/N 523-31-5431
Undercut flat head screws 4–40 thread ⅝’’ length	7	$0.06	$0.42	McMaster-Carr P/N 91099A169
Li-Ion battery (optional)	1	$9.95	$9.95	Adafruit P/N 1781
MicroUSB cable (optional)	1	$6.97	$6.97	Cdwg.com

## Synthesis

Reviewing Editor: Mark Laubach, American University Decisions are customarily a result of the Reviewing Editor and the peer reviewers coming together and discussing their recommendations until a consensus is reached. When revisions are invited, a fact-based synthesis statement explaining their decision and outlining what is needed to prepare a revision will be listed below. The following reviewer(s) agreed to reveal their identity: Jibran Khokhar, Jonathan Newman.

Two reviewers have read and commented on your manuscript. Both were enthusiastic. Please make the following essential revisions in your revised submission. The reviewers and I agree that the changes will improve the clarity of the design and robustness of the method.

1. Include an approximate build time.

2. Split Figure 1A into a larger separate figure that will make assembly easier and clearer.

3. Under comparison with current technologies, include the unique benefits of the RAT: low cost, lack of tethering to computer/wireless, lower data storage needs. Also, address this point raised by R#2, about why you did not include longer term comparisons of your method with others that have been published:

"The authors provide raw tracking data time series and compare them to existing, widely used software options for 2D animal tracking. They could statistically quantify the differences over long time periods, but I feel this is not really necessary since the differences are largely coming from small implementation and parametric details in the tracking algorithms used and there is no such thing as ground truth animal position when using video segmentation.”

4. Add the possibility of different lenses available for various behavioral needs to future improvements.

5. Please add text to address this perceived limitation of your design:

"My issues with this device is that it seems that it is a straightforward modification of existing technology (in this case, the OpenMV Cam M7). The OpenMV project has provided the camera/MCU hardware, motion tracking API, and LCD shield. The contribution of this paper on top of this existing system are a script that uses the OpenMV API for a particular tracking scenario (single animal in high contrast background), a breakout board with a reset switch and a single GPIO channel, and a 3D printed case that makes the device well suited for use in an Animal tracking scenario. What if a different tracking situation is encountered (e.g. multi subject, which would be a common situation for home-cage tracking). The script provided with this project could not handle this change, although the hardware and OpenMV API provide lots of features for using advanced machine vision to deal with this situation, and the user would have to rely on on the OpenMV API to produce a custom solution (which would remove one of the major contributions of this paper).”

6. Please add text to address this point as a potential future improvement:

"Could the TensorFlow Lite capabilities of the OpenMV camera be modified to create a Mouse Tracking network that is invariant to the background lighting conditions etc?"

7. Please consider modifications to your project to address this final point. If it is not something that can be addressed, then please summarize why in your rebuttal and add text to address this perceived limitation of the device.

"One large drawback I can envision is the lack of synchronization between individual nodes and, if I understand correctly, the lack of synchronization with a real-time clock so that data stored one each device can be aligned. Having this capability would be a large benefit to understand the effect of common-mode disturbances on behavioral data across subjects: e.g. a person entering the animal facility or the effects temperature due to HVAC lag times. The authors mention wireless communication with a central server as a potential future path, but I don’t see why this could not be included in the current design. In looking at the code, I only see relative time-stamping capabilities. I would really like to see the inclusion of a real-time clock or communication with some NTP server to get real wall-clock timestamps. Also, given the extremely low bandwidth requirements of the data, it should be quite straight forward to implement given the paths the laid out in the text and it would make dealing with files and synchronization of experimental starts for large studies much, much easier.”

## Author Response

Rebuttal document

1. Include an approximate build time.

We have now include an approximate build time of 90 minutes.

2. Split Figure 1A into a larger separate figure that will make assembly easier and clearer.

We thank the reviewer for this suggestion. We have updated Figure 1A to include this change.

3. Under comparison with current technologies, include the unique benefits of the RAT: low cost, lack of tethering to computer/wireless, lower data storage needs. Also, address this point raised by R#2, about why you did not include longer term comparisons of your method with others that have been published:

"The authors provide raw tracking data time series and compare them to existing, widely used software options for 2D animal tracking. They could statistically quantify the differences over long time periods, but I feel this is not really necessary since the differences are largely coming from small implementation and parametric details in the tracking algorithms used and there is no such thing as ground truth animal position when using video segmentation.”

While we could run the RAT side-by-side with a commercial solution for multiple days, we are not sure what this would demonstrate beyond what we show with our 1-hour tracking comparison. As the reviewer mentions, there is no “ground truth” for mouse position to compare to. In addition, assuming lighting and camera position aren’t moved, the correlations between the RAT and a commercial solution are unlikely to change with more data, as one hour already contains ∼54000 data points from the RAT. So we feel this comparison would not add much benefit.

4. Add the possibility of different lenses available for various behavioral needs to future improvements.

We thank the reviewer for this suggestion. This is in fact already possible, simply by unscrewing the lens and replacing it. We now discuss this in the paper. Certain lenses (ie: fisheye) may produce distortions that give inaccurate measures of speed at the peripheries, so we discuss this limitation as well.

5. Please add text to address this perceived limitation of your design:

"My issues with this device is that it seems that it is a straightforward modification of existing technology (in this case, the OpenMV Cam M7). The OpenMV project has provided the camera/MCU hardware, motion tracking API, and LCD shield. The contribution of this paper on top of this existing system are a script that uses the OpenMV API for a particular tracking scenario (single animal in high contrast background), a breakout board with a reset switch and a single GPIO channel, and a 3D printed case that makes the device well suited for use in an Animal tracking scenario. What if a different tracking situation is encountered (e.g. multi subject, which would be a common situation for home-cage tracking). The script provided with this project could not handle this change, although the hardware and OpenMV API provide lots of features for using advanced machine vision to deal with this situation, and the user would have to rely on on the OpenMV API to produce a custom solution (which would remove one of the major contributions of this paper).”

While we agree that the OpenMV camera did the heavy lifting here (and we cite them repeatedly and do not try to minimize this!), we believe our unique contribution is in the application of their camera to a new use case, providing the electronics modifications and instructions to improve workflow for a mouse-tracking application, and most importantly providing rigorous testing data to demonstrate that the OpenMV camera can perform well in this tracking situation. By analogy, many people build devices based on the Arduino hardware ecosystem. Despite not creating their own microcontroller boards, people provide value by devising unique use cases and testing their performance in these applications. We believe what we have done with the OpenMV camera is very analogous to this, and our value is in showing that animal tracking can be accomplished on an embedded microcontroller like the OpenMV camera (at a time when most of the effort in rodent machine vision tracking is moving towards very powerful computers and GPUs), as well as providing detailed instructions and code for others to see how we did it and how they can modify and build on what we’ve done.

With respect to multiple animal tracking, this is a very difficult problem in machine vision, and we do not think the OpenMV features would be able to keep track of two black mice in a box. There aren’t good solutions for this problem even in stand-alone software packages with access to a full-fledged PC and GPU. For example, this is one of the stated future goals of DeepLabCut, a program that requires many magnitudes more computational resources than the OpenMV camera to run, and even still does not run in real-time. Given the computational difficulty of this problem, we think it unlikely to be accomplished with the OpenMV hardware, and we now expound on this point and our opinion of the state of technology in the discussion.

6. Please add text to address this point as a potential future improvement:

"Could the TensorFlow Lite capabilities of the OpenMV camera be modified to create a Mouse Tracking network that is invariant to the background lighting conditions etc?"

In theory this could be possible, although this is not possible with the version of the OpenMV camera we used (the M7). Since submitting our first draft, the OpenMV team did introduce an upgraded camera (the H7), which can theoretically run a version of TensorFlow Lite. However, this has not yet been implemented on the H7 camera, it is instead a theoretical possibility given the chipsets they used. The developers of the camera hope to have it implemented soon but give no timeline. Given this state, the answer to this question is currently no, however it is likely to be possible on similar solutions in the future. We have added a line discussing deep learning mouse tracking network to the discussion.

The reviewer’s point about background lighting variation is well taken though, and in line with our goal to create a useful device we implemented an alternative solution in our code, which was to create an “auto-thresholding” procedure, where the camera checks the image statistics every ∼4 seconds, and if the mean brightness changes beyond 1 standard deviation in either direction (for instance, if someone turns on an overhead room light), it “re-thresholds” to keep track of the mouse. In our experience this approach was robust against the most common forms of background lighting variation a user would likely encounter in a laboratory setting. Again, all of our code is open-source, so if a user had a particularly challenging lighting and tracking situation they could use our code as a start, and optimize it themselves to develop a custom solution for their needs.

7. Please consider modifications to your project to address this final point. If it is not something that can be addressed, then please summarize why in your rebuttal and add text to address this perceived limitation of the device.

"One large drawback I can envision is the lack of synchronization between individual nodes and, if I understand correctly, the lack of synchronization with a real-time clock so that data stored one each device can be aligned. Having this capability would be a large benefit to understand the effect of common-mode disturbances on behavioral data across subjects: e.g. a person entering the animal facility or the effects temperature due to HVAC lag times. The authors mention wireless communication with a central server as a potential future path, but I don’t see why this could not be included in the current design. In looking at the code, I only see relative time-stamping capabilities. I would really like to see the inclusion of a real-time clock or communication with some NTP server to get real wall-clock timestamps. Also, given the extremely low bandwidth requirements of the data, it should be quite straight forward to implement given the paths the laid out in the text and it would make dealing with files and synchronization of experimental starts for large studies much, much easier.”

We thank the reviewer for this comment and agree that a way to synchronize timing would greatly improve the functionality of this device, especially as we envision it being cheap and simple enough to use that labs might build several of them and run them simultaneously. The authors are correct that the prior device did not include a real-time-clock (RTC) so it logged data all in relative time from the start of a session. We agree that the addition of a real “timestamp” would greatly improve the device.

Based on our experience with creating WIFI-enabled microcontroller solutions in the past, we decided against including a WIFI connection for receiving the timestamp. While in theory this is a viable approach, in practice the WIFI connection can be a source of frustration with microcontroller boards, particularly when trying to connect to secure enterprise networks that exist in many Universities (or in our case the Federal Government which has extremely restrictive WIFI policies). Microcontrollers often do not have the ability to navigate advanced WIFI security protocols, and even when they do it can be cumbersome to set them up.

Therefore, we decided to take the reviewer’s other suggestion to include an on-board battery backed-up real-time clock (RTC). We added a hardware connection to the PCB, as well as software support for the Adafruit DS3231 Precision RTC. This required a revision to the PCB, as well as modification to the code to set the RTC and recall RTC timestamps. The data files now include the RTC timestamp on every line, to allow millisecond precision synchronization between devices. We include a new visualization of the build, including the new PCB and RTC module in Figure 1.

For the reviewer’s info we provide a screenshot of the new datafiles, showing the time-stamping of every video frame.
